# Aging-Related Structural and Functional Changes in Cerebral Arteries: Caloric Restriction (CR) Intervention

**Published:** 2021-09-07

**Authors:** Maurizio Mandalà, Marilyn J. Cipolla

**Affiliations:** 1 Department of Biology, Ecology and Earth Sciences, University of Calabria, Arcavacata di Rende (CS), Italy; 2 Department of Obstetrics, Gynecology and Reproductive Science, University of Vermont, Burlington, VT 05405, USA; 3 Department of Neurological Sciences, University of Vermont Larner College of Medicine, Burlington, VT, USA

**Keywords:** Aging, Caloric restriction, Cerebrovascular disease, Smooth muscle cells, Endothelial cell

## Abstract

Cerebral arteries play a crucial role in the regulation of blood flow to the brain to satisfy the demand of oxygen and glucose for proper function of the organ. Physiological cerebral blood flow (CBF) is maintained within a normal range in response to changes in blood pressure a mechanism named Cerebral Blood Flow Auto Regulation (CBFAR). Structure and function of cerebral arteries have an important impact on CBFAR. Several studies in human and animals have showed significant morphological and functional changes in cerebral vessels of aged brain associated with a reduced CBF which is also impaired in cerebrovascular pathology linked to brain diseases. Interestingly, one new emergent aspect is the lifelong Calorie Restriction (CR) as a potential intervention to prevent age-related cerebral artery changes and preserve the health of aging brain. This review summarizes the recent literature on the effects of aging on cerebral artery structure and function and the potential of CR as opportunities for prevention and treatment.

## CEREBRAL VESSELS IN AGING HUMANS

Significant structural and functional changes occur in the cerebrovascular system with advancing age, even in apparently healthy individuals. Morphological analysis of cerebral arteries from aging human brain have shown abnormalities in both large (pial) and small (parenchymal) arteries and arterioles [1–3]. Arteries of the Circle of Willis from elderly brains showed abnormalities, including cell vascular wall hypoplasia, anatomical variations, and increased incidence of aneurysms with little association with neurodegenerative and cerebrovascular disorders [1]. Further, small arteries of the aged brain appear as elongated and tortuous vessels with narrowed lumens and thickened vessel walls [4,5]. The main vascular risk factor from cerebral blood vessel narrowing is brain hypoperfusion/ischemia due to; these changes are associated to a significant decrease reduction of cerebral blood flow (CBF). Studies in aged human and animal brains have shown a significant reduction of 20–40% compared with young brains [6,7]. Ischemic attacks are very frequent in the elderly and the global burden of stroke is increasing dramatically. Current epidemiological data indicate that 16.9 million people suffer a stroke each year. An occlusion of brain arteries constitutes a major risk for disability or death. The cerebral hypoperfusion may compromise cognitive function and may be associated with Alzheimer’s pathology by rapid beta-amyloid deposition in brain capillaries [8]. The genesis of these changes in both large and small cerebral arteries is not fully understood; however, some suggestions include:

- Depletion of vascular myocytes from the tunica media [4,9,10],
- A switch of myocyte phenotype from the contractile to the synthetic phenotype with loss of contractile properties due to alterations of the vascular wall extracellular matrix [11]
- Endothelial dysfunction including thinning and reduction of endothelial derived relaxation factors [12,13].

Small cerebral arteries contribute to CBFAR and age-related impairment, including Cerebral Small Vessel Disease (CSVD) that is strongly associated with stroke, hemorrhage and cognitive decline [2, 14]. In addition, advancing age decreases brain vessel density (rarefaction) [15]. This combined with aging-related changes in cerebral arteries exacerbate the chronic hypoperfusion and decline in cognitive functions. Further, chronic hypertension that is frequent in aged individuals can worsen the normal physiological processes of aging-related cerebral arteries through several mechanisms including increased pulse pressure [16].

### Aging affects cerebral vessels biomechanical properties

In human and animals, changes of cerebral artery biomechanical properties are associated with aging. Aging induces changes in the structure of mouse Posterior Cerebral Arteries (PCA) and in Parenchymal Arterioles (PAs), demonstrating changes in large arteries and the microcirculation [15]. In particular, aging increased lumen diameter and cross section (outward remodeling) of PCA and decreased artery stiffness (reduced distensibility). The stiffness can be explained by changes in the vascular wall composition and organization. For example, aging increases collagen and reduces the elastin deposition in the vascular wall [15,17] due to an increase of matrix metalloproteases expression in advancing age [18]. In cerebral arteries of aged humans, a reduced distensibility and loss of compliance due to fragmentation and change in organization of elastin fiber has also been shown [19].

In PAs of mice, wall thickness and cross-sectional area were larger in old (22–24 months) vs. young animals (3–5 months) [15] with an increase of the ratio of the collagen to elastin and concurrent reduction of the CBF [7]. This suggests that age- related changes in the cerebral vascular structure and distensibility may be related to diminished brain blood flow. Also in aged rats (24–27 months) diameter and distensibility of PAs were reduced [20]. Increased arterial stiffness is a hallmark of artery dysfunction and a biomarker of cardiovascular disease [21].

### Cerebral artery functional changes with advancing age

Stable CBF in healthy brain is accomplished by the normal function of the cerebral arteries that dilate or constrict in response to physiological chemical messengers and changes in cerebral perfusion pressure. Aging promotes functional alterations in the cerebral vasculature [22] with high risk for cerebral vascular diseases such as stroke and dementia [23]. In human middle cerebral artery, impairment of reactivity (vasodilation and vasoconstriction) was found in elderly individuals [24]. Also in rat basilar arteries, advancing age reduced contractility to endothelin-1 and relaxation to acetylcholine [25]. Further, aging impairs myogenic adaptation to pulsatile intraluminal pressure (26). Interestingly, aging appears to affect cerebral artery function differentially in different vascular segments, starting in the pial arterial network months before microcirculatory dysfunction and global changes in CBF are detectable [26].

Although limited in number, some studies have investigated the molecular mechanisms underlying aging-impaired cerebral vascular reactivity and suggest age-related changes occurred in both vascular Smooth Muscle Cells (SMCs) and Endothelial Cells (ECs). In SMCs, aging disrupted calcium (Ca2+) signaling by modification of gene expression of ion channels and pumps involved in Ca2+ signaling implicated in the regulation of vascular reactivity [27]. In addition, aging also affects the large- conductance calcium-activated potassium (BK) channels that are key regulator of cerebral arterial tone in a sex-specific manner [28]. In particular, aging decreased BK channel currents in cerebral vascular SMCs from female while increased it in male rats [28].

Aging also affects the function of ECs, the inner cellular layer of cerebral blood vessels that balances coordination of vasodilation and vasoconstriction for optimal blood flow distribution in the brain. Aging reduces endothelial nitric oxide synthase (eNOS) signaling by potential mediators of vascular dysfunction such as Reactive Oxygen Species (ROS), Poly(ADP-ribose) Polymerase (PARP) [12] and Rho kinase (ROCK) that are potential mediators of vascular dysfunction [29,30]. In addition, Endothelium-Derived Hyperpolarization (EDH) is reduced by aging due to diminished function of Gq -Protein-Coupled Receptors (GPCRs) and K+ channels [13]. Further, a role for angiotensin II (ANG II) and the angiotensin Type 1 (AT1) receptor [12] in age-related cerebral vascular dysfunction has been suggested as well as for both endothelin-1 receptors, ETA and ETB which respond to endogenous endothelins to regulate blood vessel diameter [31].

**Figure 1:**
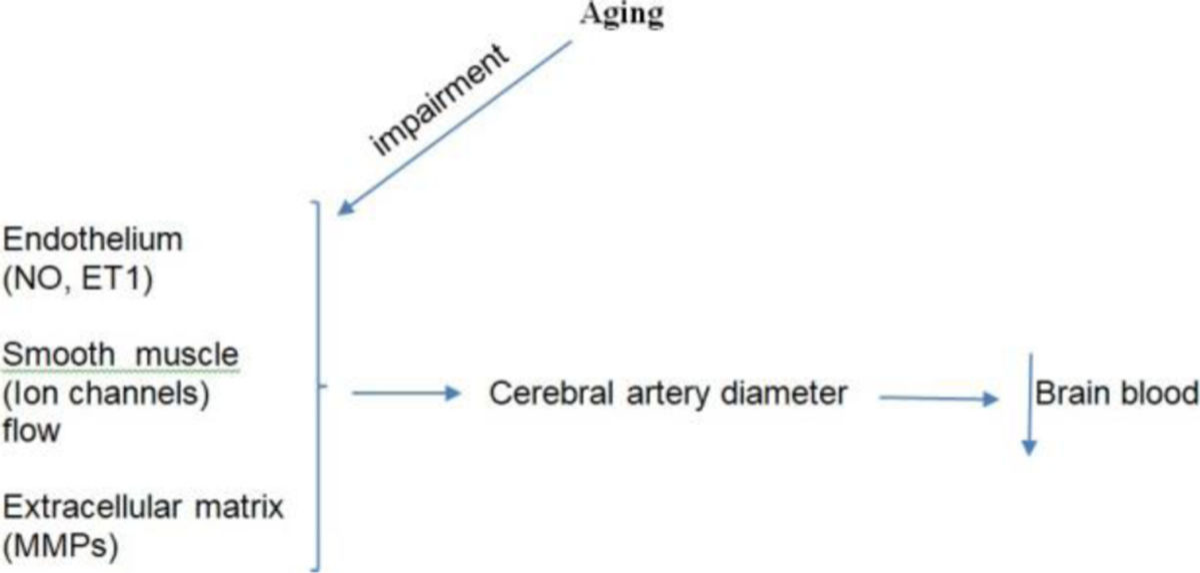
Aging impairs crucial cerebral artery vasoregulatory mechanism through.

In summary, aging may impair local vasoregulatory mechanisms intrinsic to the vascular wall of cerebral arteries, (Figure 1) leading to increased micro vascular injury and high risk of brain infarctions, ischemic attacks and white matter lesions in older individuals [32].

- Altered endothelium-dependent mediators largely attributed to nitric oxide (NO) and endothelin one-1 (ET1) 2)
- Changes in ion channels expressedion and activity in both endothelial and vascular smooth muscle cells and 3)
- Activation of Matrix Metalloproteinase (MMPs) critical for the maintenance of the extracellular matrix organization that impacts vessel stiffness. Together, this Aging-related impairments compromise the regulation of cerebral artery diameter with consequent decrease of CBF blood flow to brain.

## DISCUSSION

### Food restriction on age-related cerebral vascular structure and function

Several lines of evidence suggest that overeating is a challenge in public health. Therefore, considerable effort has been devoted to determine if Caloric Restriction (CR)-a chronic and coordinated reduction in food intake without causing malnutrition-could prevent the pathophysiological consequences of overeating. Numerous studies mainly in rat and mouse showed that animals with food intake restriction by 30–60% live longer and have fewer age-associated diseases compared to animals fed ad libitum [33]. The health benefit of CR has been extended to various species including humans [34] and the most cited mechanism is the attenuation of oxidative damage which has been shown to slow aging, protect against much age-related pathology, and extend the lifespan in various species [35]. However, although CR has been widely examined (70 years that it has been studied) as a preventative strategy against aging and various diseases, its effects on aged cerebral arteries are largely unknown. Few and recent studies have showed that CR could reverse the onset of age-related cerebrovascular disease and cognitive decline. A study in mouse middle cerebral arteries showed that age-related endothelial dysfunction was rescued by lifelong CR that prevented the decline in NO bioavailability and the onset of NADPH oxidase-mediated oxidative stress [36]. In addition, CR enhanced neurovascular functions with preserved cognitive function and reduced anxiety of aging mice [37]. In this study, the mammalian Target of Rapamycin (mTOR) pathway was shown to be involved. In particular, CR inhibited mTOR activity that increased eNOS and subsequently increased CBF [37]. In aged rats (24 months), lifelong CR increased vascular tone of PCA in response to intraluminal pressure as well as constriction to Potassium Chloride (KCl) tested in the range of concentrations 25–80 mm [38]. This suggests that CR reverses the vascular tone reduction due to aging with the protective role to prevent cerebral injuries in the microvasculature. In addition, CR also increased PCA distensibility [38], improved cerebral artery compliance, with a positive impact on CBFAR. Although, in this study the authors did not demonstrate the underlying mechanism, we assumed that CR could reverse the aging-related changes in the ratio of elastin and collagen in the vascular wall amount through regulation of the matrix metalloproteinase secretion and activity.

## CONCLUSION

This brief review underlying the importance of the cerebral vessel integrity as a major determinant of a healthy functional brain. Age-induced vascular dysfunction compromises CBFAR and increases the risk of cerebrovascular disease such as stroke and dementia which are major causes of mortality and morbidity in the elderly. Evidence points to the vascular system as a potential therapeutic target for cerebral vascular diseases in aging. Therefore, it is important to identify interventions that could prevent vascular impairments that occur with advancing age. Lifelong CR reduces the onset of age-related cerebral vascular changes to sustain cerebral vascular integrity critical to preserving brain function in aging and reducing the risk of age- related cerebrovascular disorders.

## LIMITATIONS FUTURE RESEARCH

The majority of studies on CR in the literature were carried out on rats or mice, with few studies in human. In addition, most of the studies were conducted in male animals and therefore sex differences are not known. Therefore, future studies are needed to investigate the interactions of age and sex on cerebral vasculature properties in aging and CR. In addition, when age- related cerebrovascular dysfunction starts across the lifespan is also an important area of investigation that may allow early intervention to prevent or postpone the onset of cerebrovascular disease especially with CR. Furthermore, the underlying mechanisms by which CR prevents aging-related cerebral vasculature changes are important to understand. Lastly, future studies should take into account genetic background to identify if personalized caloric restriction would be useful to slow brain aging and to prevent cerebrovascular disease.
